# Investigation Into the Variations in the Interactions Between Different Selenium Sources and Gut Microbiota

**DOI:** 10.1002/fsn3.71934

**Published:** 2026-06-04

**Authors:** Meiyu Yuan, Yihong Li, Zixuan Zhou, Wanting Dai, Xian Cui, Shuang Bi, Xiaoxiao Song

**Affiliations:** ^1^ State Key Laboratory of Food Science and Resources, China‐Canada Joint Lab of Food Science and Technology (Nanchang), Key Laboratory of Bioactive Polysaccharides of Jiangxi Province Nanchang University Nanchang China; ^2^ School of Food and Health Beijing Technology& Business University Beijing China

**Keywords:** gut microbiota, nanoparticles, polysaccharide, selenium

## Abstract

Selenium (Se) exists in various chemical forms, including inorganic Se, organic Se, and Se nanoparticles (SeNPs). However, the mechanisms underlying the interaction between Se and gut microbiota remain unclear. This study systematically evaluated the effects of different polysaccharide‐stabilized Se nanoparticles (PS‐SeNPs) using an in vitro colonic fermentation model. The results indicate that the molecular properties of polysaccharides (PS) are key factors influencing the gut microbiota‐modulating effects of PS‐SeNPs. Specifically, high molecular weight and positively charged PS enhance the microbial utilization of SeNPs. Chitosan‐based Se nanoparticles (CS‐Se) and chitosan quaternary ammonium salt‐based Se nanoparticles (HACC‐Se) can specifically enrich beneficial bacterial communities, such as *Lactobacillus* and *Bacteroidales*, which possess both acid production and Se metabolism capabilities. This enrichment significantly promotes the accumulation of short‐chain fatty acids and the generation of selenocystine (SeCys_2_) and Se‐Methylselenocysteine (MeSeCys). Metabolomics analysis further showed significant activation of amino acid biosynthetic pathways. Chitosan oligosaccharide‐based Se nanoparticles (COS‐Se) and carboxymethyl cellulose‐based Se nanoparticles (CMC9‐Se) can enrich beneficial microorganisms. However, their bioavailability and microbial assimilation efficiency remain relatively limited. The H_2_SeO_3_ group significantly promoted the proliferation of microorganisms such as *Enterobacteriaceae, Escherichia‐Shigella, and Klebsiella*. Our findings could improve understanding of the interaction between Se and gut microbiota.

## Graphical abstract

Molecular weight and charge of polysaccharides dictate the microbial utilization efficiency of selenium nanoparticles. Chitosan‐based selenium nanoparticles optimize Se metabolism and gut health via specific microbial enrichment.

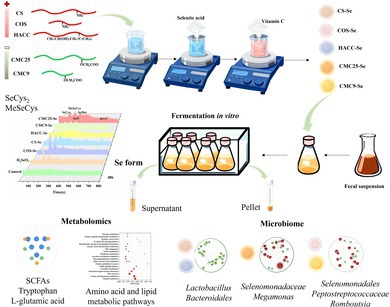

## 1 Introduction

Selenium (Se) is an essential micronutrient for mammals, crucial for maintaining redox balance, antioxidant defense, and immune regulation (Dai et al. 2025; Song, Chen, Sun, et al. 2020; Song, Chen, Zhao, et al. 2020). Common selenium‐containing compounds include inorganic Se (Se (VI) and Se (IV)), organic Se (SeCys_2_, MeSeCys, and SeMet), and Se nanoparticles (SeNPs). Recent studies have revealed a complex bidirectional interaction between selenium and the gut microbiota. On one hand, approximately 25% of gut microbiota encode selenoproteins, which not only compete with the host for ingested selenium but also transform selenium into different forms through their own metabolic processes (Gangadoo et al. 2019; Karunakar et al. 2025), which directly affect the bioavailability and toxicity of selenium (Zhang et al. 2025). On the other hand, the gut microbiota can reduce inorganic selenium to elemental selenium nanoparticles (SeNPs) or convert it into selenoamino acids and selenides, which in turn can influence the colonization and composition of the gut microbial community (Wang, Zhong, et al. 2025). A study showed that SeMet, MeSeCys, and SeCys_2_ increased alpha diversity and the abundance of *Bacteroidota*, whereas selenite increased the F/B ratio and decreased the abundance of *Bacteroidota* (Liang et al. 2024). Among the numerous forms of selenium, SeNPs have garnered significant attention due to their superior bioavailability and reduced toxicity.

However, practical application of SeNPs is often limited by issues such as aggregation and instability. To address these challenges, surface modification with natural polysaccharides (PS) has been widely adopted. Recent studies have shown that PS, which contain abundant hydroxyl groups, can bind to Se to form PS‐SeNPs. These PS enhance nanoparticle stability. Further research has found that the molecular properties of PS, such as molecular weight, charge properties, and functional group modifications, are the core factors determining the biological behavior of PS‐SeNPs (Song et al. 2021; Zhang et al. 2022). Research has shown that polysaccharides can significantly enhance the colonic retention properties of SeNPs, thereby prolonging their contact time with gut microbiota (Fang et al. 2025). This characteristic further endows PS SeNPs with the ability to regulate specific bacterial populations, enabling them to selectively increase the abundance of beneficial bacteria such as *Bacteroides, Bifidobacterium*, and *Lactobacillus*, and to promote the production of short‐chain fatty acids (SCFAs) (Bamigbade et al. 2025). However, most existing studies have primarily considered PS as physical stabilizers for SeNPs, overlooking their molecular properties (such as molecular weight, functional groups, and charge) in regulating the interaction between PS‐SeNPs and gut microbiota, especially their potential impact on Se form transformation and host metabolic functions. At present, understanding of the interaction between SeNPs and gut microbiota is still limited, especially lacking systematic exploration from the perspective of polysaccharide properties. Therefore, it is necessary to further analyze the specific regulatory effects of SeNPs stabilized by PS with different molecular characteristics on the structure and metabolic function of gut microbiota.

In this study, we used chitosan oligosaccharide (COS), chitosan (CS), chitosan quaternary ammonium salt (HACC), and carboxymethyl cellulose (CMC) with different molecular weights—as templates to prepare PS‐SeNPs. Then, we adopted an in vitro colon fermentation model combined with 16S rRNA sequencing and non‐targeted metabolomics, and investigated the differences in bacterial composition, Se form and metabolism induced by PS‐stabilized SeNPs with varying characteristics, and additionally compared the biological effects with traditional organic Se and inorganic Se.

## 2 Materials and Methods

### 2.1 Materials and Reagents

COS, CS, CMC9, CMC25, and HACC were purchased from Shanghai Yuanye Biotechnology Co. Ltd., China. CMC was purchased from Shanghai Macklin Bio‐chemical Technology Co. Ltd., China. Vitamin C, selenite acid, and selenomethionine (SeMet) were purchased from Shanghai Aladdin Biochemical Technology Co. Ltd., China. The molecular weights of COS, CS, HACC, CMC9, and CMC25 are 2, 62, 91, 9, and 25 kDa. SCFA standards (acetic acid, propionic acid, isobutyric acid, butyric acid, and isovaleric acid) were purchased from Sigma Corporation (USA).

### 2.2 Preparation of PS‐SeNPs

To prepare polysaccharide‐selenium nanoparticles, each polysaccharide (COS, CS, CMC9, CMC25, or HACC; 10 mg) was dissolved in 10 mL of 0.2% acetic acid under stirring. After 4 h, H_2_SeO_3_ was added to reach 2 or 4 mmol/L, followed 4 h later by vitamin C to 4 or 8 mmol/L, yielding the corresponding low‐ (PS‐SeNPs‐L) or high‐concentration (PS‐SeNPs‐H) nanoparticles, respectively; the resulting dispersion was then dialyzed to remove unbound H_2_SeO_3_ and VC, and the sample was freeze‐dried for future use (Dai et al. 2025).

In parallel, control solutions containing selenium in forms equivalent to the selenium content in PS‐SeNPs‐L and PS‐SeNPs‐H were prepared by directly dissolving selenomethionine (SeMet) and H_2_SeO_3_ in deionized water. These solutions were designated as SeMet‐L, SeMet‐H, H_2_SeO_3_‐L, and H_2_SeO_3_‐H.

### 2.3 Particle Size and Zeta Potential

The particle size and zeta potential of the different nanoparticles were determined using a Zetasizer Nano ZS 90 (Malvern Instruments, UK). Particle Size Measurement: Before measurement, the SeNPs suspension was equilibrated at 25°C for 2 min. SeNPs were moved into a colorimetric dish with an optical path of 1 cm for the size measurement. The measurement temperature was maintained at 25°C, and the refractive index of the dispersant was set to 1.33. Each sample was measured independently at least three times, and the final results were reported as the average particle size (Z‐average).

Zeta Potential Measurement: Take an appropriate amount of the SeNPs suspension and inject it into the potential cell. Allow the sample to equilibrate at 25°C for 2 min before measurement. Each sample should be measured at least three times to ensure accuracy (Jiang et al. 2025).

### 2.4 Fourier Transform Infrared (FT‐IR) Spectroscopy

The infrared spectra of PS and PS‐SeNPs were measured using the KBr pellet method. Pure KBr was dried for 24 h, after which 150 mg was accurately weighed and mixed with 1 mg of the sample. The mixture was transferred to a tableting mold and maintained under a pressure of 10 MPa for 2 min to produce a transparent sheet. The sheet was then scanned using an FTIR spectrometer (IS50, Thermo Fisher Scientific, USA) over a wavenumber range of 4000–400 cm^−1^. Each sample was prepared and measured three times. After automatic baseline correction and normalization of the obtained spectra, the characteristic absorption peaks were analyzed using OMNIC software (Tang et al. 2025; Yuan et al. 2025).

### 2.5 Water Droplet Contact Angle

A contact angle measuring instrument was used to determine the hydrophilicity and hydrophobicity of PS‐SeNPs. Take 100 mg of the PS‐SeNPs sample and press it into a flat and compact disc with a diameter of 1 cm. Place the film on the sample stage and add deionized water using a micropipette. Wait until the droplet stabilizes, then measure the static contact angle using the goniometric or circle‐fitting method. Three tablets were prepared for each sample, and at least three locations on each tablet were randomly selected for measurement. The results are expressed as the mean ± standard deviation.

### 2.6 Atomic Force Microscope (AFM)

Take an appropriate amount of PS‐SeNPs suspension and dilute it with deionized water. Using a pipette, place 10 μL drops onto the surface of a mica sheet. Allow the drops to settle naturally for about 30 s, then gently remove any excess liquid from the edges with filter paper. Finally, place the sample in a clean environment to dry at room temperature, ensuring complete evaporation of moisture. Subsequently, the deposited film was scanned over a 1 × 1 square micron area. AFM (Bruker Malaysia Sdn Bhd, Germany) captures the experimental data in digital form as sets of *x*, *y*, and *z* values and reconstructs the morphology of a sample in real time on a computer screen (Mishra et al. 2025).

### 2.7 In vitro Fecal Fermentation

An in vitro simulated colonic fermentation model inoculated with human fecal microbiota was employed to evaluate the fermentation characteristics of the samples. Specifically, the effects of the following sample groups were assessed at 24 and 48 h: PS, PS‐Se‐L, PS‐Se‐H, SeMet‐L, SeMet‐H, H_2_SeO_3_‐L, H_2_SeO_3_‐H.

The in vitro fermentation was performed based on a previous method with some modifications. Briefly, fresh fecal samples were collected from four healthy human donors who did not use any antibiotics and probiotics for at least 3 months. The experimental procedures followed the Guidelines of the Experimental Ethical Committee of College of Food Science, Nanchang University. Equal amounts of feces from each donor were mixed and then diluted with phosphate buffer. After filtering through four layers of gauze, the filtrate was obtained and subsequently added to sterilized medium (containing Vitamin k1 and heme chloride). The fecal solution was mixed with PS, PS‐Se‐L, PS‐Se‐H, SeMet‐L, SeMet‐H, H_2_SeO_3_‐L, and H_2_SeO_3_‐H. The low concentration groups contained 10 μg/mL of selenium, while the high concentration groups contained 20 μg/mL. Each mixture was fermented anaerobically for 24 and 48 h, respectively.

The selenium concentration chosen for this experiment was primarily based on previous literature reports. Research has demonstrated that within this concentration range, selenium enrichment can effectively modulate the gut microbiota structure and promote the production of short‐chain fatty acids (SCFAs). Additionally, establishing low and high concentration gradients allows for the observation of selenium concentration‐dependent effects on microbial community regulation (Ji et al. 2022; Liang et al. 2024). Each mixture was fermented for 24 and 48 h, respectively, under the anaerobic condition. Following each fermentation at the above different intervals, the samples were frozen at −80°C prior to subsequent 16S rRNA gene sequencing and determination of SCFAs.

### 2.8 Determination of Short‐Chain Fatty Acids (SCFAs) Contents and pH Value

PS, PS‐Se‐L, PS‐Se‐H, SeMet‐L, SeMet‐H, H_2_SeO_3_‐L and H_2_SeO_3_‐H fermentation samples were centrifuged for 10 min at 10,000 ***g*** to obtain supernatants for the determination of SCFAs. The pH of the supernatant was immediately measured using a calibrated pH meter. For the determination of SCFAs, the supernatant (500 μL) was mixed with 10% H_2_SO_4_ (10 μL) and ethyl ether (800 μL). The SCFAs in the mixture were analyzed by gas chromatography. Gas chromatograph measurement parameters: injection volume of 1 μL; split ratio of 20:1; carrier gas: nitrogen; injection port temperature: 220°C; column flow rate: 2 mL/min; initial temperature: 60°C, maintained for 3 min, then increased at a rate of 10°C/min to 190°C and maintained for 15 min; detector temperature: 275°C; chromatographic column used: Nukol (30 m × 0.53 mm × 0.5 μm).

### 2.9 Determination of Se Form and Se Content

Appropriate amounts of the samples (PS‐Se‐L, SeMet‐L, and H_2_SeO_3_‐L fermentation broths at 24 and 48 h) were pretreated by enzymatic hydrolysis for pretreatment. Briefly, the sample was dissolved in 15 mL Tris–HCl (pH = 8.5) with the addition of 5% alkaline protease, 5% trypsin, and 5% protease K. The reaction was carried out at 45°C for 4 h. HPLC‐ICP‐MS was used to detect different Se species in the samples. Total selenium concentrations were determined by inductively coupled plasma mass spectrometry (ICP‐MS; Agilent 7700). Selenium was measured at m/z 78 using the octopole reaction system (ORS) with hydrogen (3.5 mL min^−1^). Indium (200 μg L^−1^) was introduced online as an internal standard. Quality control was performed using the certified reference material TORT‐2. To enhance the selenium signal and compensate for carbon‐related matrix effects, CO_2_ (0.15 L min^−1^, 1% in Ar) was added online between the spray chamber and the torch (Tangjaidee et al. 2022; Zhang et al. 2025).

To accurately evaluate the regulatory effects of different selenium sources on gut microbiota, this study selected a PS‐SeNPs‐L group (which exhibits good biocompatibility) and a 24 h fermentation time point (when the levels of SCFAs and key selenium metabolites were high) for subsequent analysis. The focus was on examining the microbial changes and metabolomic characteristics of PS‐SeNPs‐L at 24 h of fermentation.

### 2.10 Microbial Community Diversity Analysis

Total genomic DNA was extracted from the microbiota of the samples: PS‐Se‐L, SeMet‐L, H_2_SeO_3_‐L, and the PS fermentation broths at 24 and 48 h. Total DNA of microbiota was extracted using QIAamp DNA Stool Mini Kit (QIAGEN, Hilden, Germany). The primer set of 338F (ACTCCTACGGGAGGCAGCAG) and 806R (GGACTACHVGGGTWTCTAAT) was used to amplify the V3‐V4 region of the 16S rRNA gene via polymerase chain reaction (PCR). The amplification product was sequenced on Illumina MiSeq PE300 platform. The sequencing data was analyzed on the Majorbio Cloud platform. Raw data were quality‐filtered using Trimmomatic. UPARSE (v7.1) clustered operational taxonomic units (OTUs) at 97% similarity, with chimeras removed by UCHIME. Taxonomic annotation against Silva database (v138) was performed using RDP classifier (70% confidence).

### 2.11 Metabolomics Analysis

Metabolomics analysis was performed on the following samples: PS‐Se‐L, SeMet‐L, H_2_SeO_3_‐L, and PS fermentation broth. Metabolomics analysis was performed using liquid chromatography–tandem mass spectrometry (LC–MS/MS) on a UHPLC‐Q Exactive HF‐X platform, with detailed methodology provided in the Supporting Information.

### 2.12 Statistical Analysis

All the experiments were repeated three times and data were presented as mean ± SD. Differences between groups were evaluated using one‑way ANOVA followed by a post‑hoc multiple comparison test in which *p* < 0.05 was considered to indicate statistical significance. Statistical analysis was performed using SPSS 26.0 software (SPSS, Chicago, IL, USA) and Origin 2024 software (Origin Lab, USA) was used for the graphical analysis.

## 3 Results and Discussion

### 3.1 Characterization of PS‐SeNPs

#### 3.1.1 Particle Size, Zeta Potential, AFM, and Water Droplet Contact Angle

**FIGURE 1:**
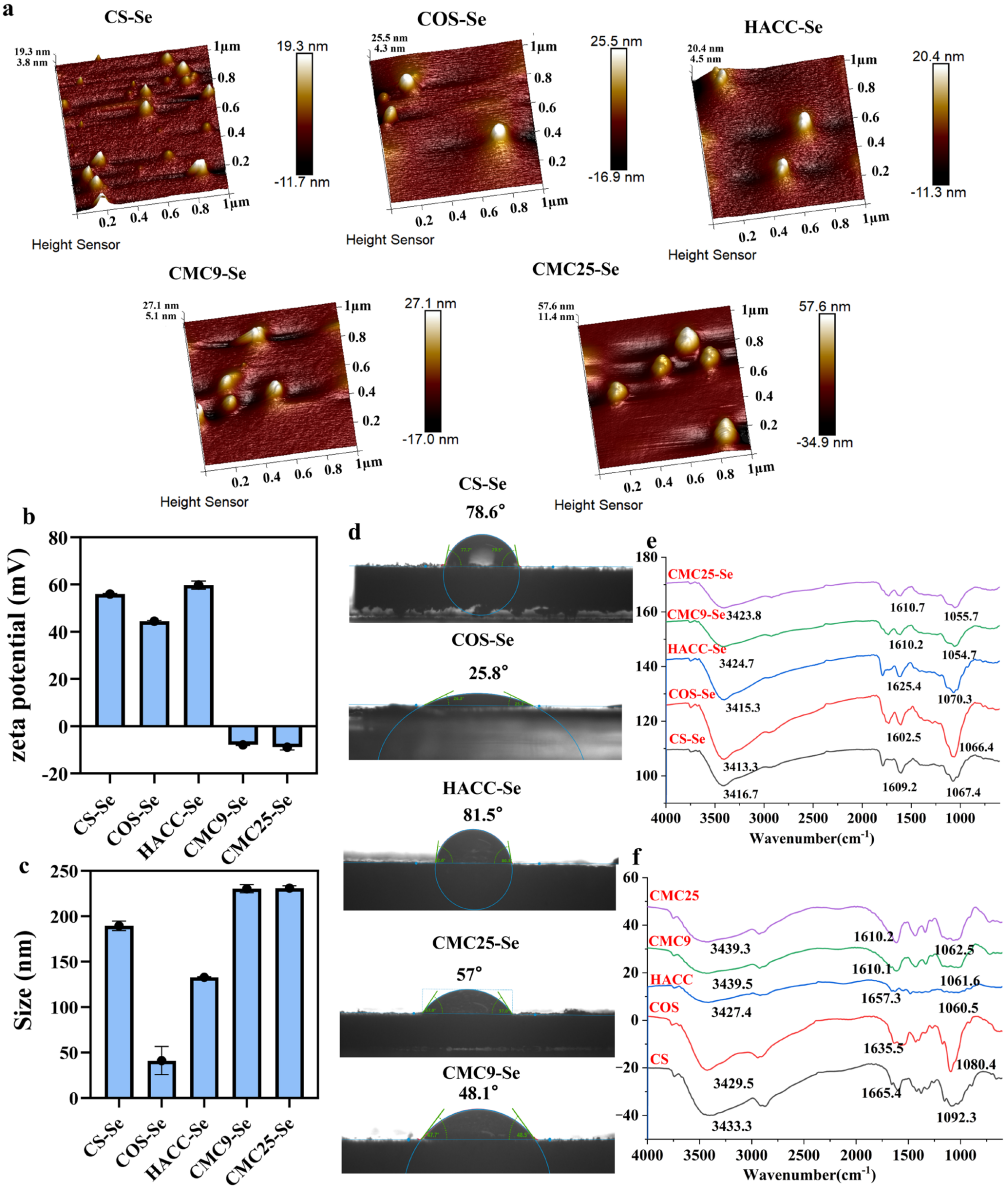
(a) 3D topographic AFM image. (b) Zeta potentials of PS‐SeNPs. (c) Particle size. (d) Contact angle of PS‐SeNPs. The FTIR of PS‐SeNPs (e) and PS (f).

The AFM analysis revealed that the particles are spherical, smooth, and have a surface size of approximately 300 nm (Figure 1a). These PS‐SeNPs exhibit similar structures in AFM morphology; however, differences in particle distribution and surface height variations are observed. CS‐Se, HACC‐Se, and others have lower and relatively concentrated heights, around 30–50 nm, while CMC25‐Se has a higher height which may be related to the type of polysaccharide, corroborating the findings of previous studies (Ahmad et al. 2025).

The particle sizes of CMC25‐Se and CMC9‐Se were significantly larger than those of the other SeNPs; among them, COS‐Se exhibited the smallest particle size. Specifically, CMC25‐Se = CMC9‐Se > CS‐Se > HACC‐Se > COS‐Se (Figure 1c). The zeta potentials on the surfaces of CMC25‐Se and CMC9‐Se are negative. In contrast, CS‐Se, HACC‐Se, and COS‐Se exhibit positive charges due to amino protonation in chitosan derivatives (Figure 1b).

The hydrophobicity of PS‐SeNPs in descending order was as follows: HACC‐Se, CS‐Se, CMC25‐Se, CMC9‐Se, and COS‐Se. Among them, COS‐Se exhibited the smallest contact angle, indicating stronger hydrophilicity and weaker hydrophobicity (Figure 1d).

#### 3.1.2 Fourier Transform Infrared Spectroscopy

To investigate the chemical binding modes between different PS and Se, FTIR was used to characterize PS and its modified PS‐SeNPs. The peak at 3400–3450 cm^−1^ corresponds to the stretching vibration of O–H (Dai et al. 2025), and the peak at 1000–1200 cm^−1^ corresponds to the stretching vibration of glycosidic bonds C–O–H and C–O–C (Abbas et al. 2025). The peak at 1700–1600 cm^−1^ corresponds to N‐H stretching vibration (Zeng et al. 2025). As shown in Figure 1e,f, the O–H stretching vibrations of CS‐Se, COS‐Se, and HACC‐Se all underwent blue or red shifts. Compared with PS, the C–O–H and C–O–C vibration peaks in all PS‐SeNPs samples also showed varying degrees of red or blue shift. In addition, the N–H vibration peaks in COS‐Se, CS‐Se, and HACC‐Se also showed significant shifts. Therefore, COS, CS, and HACC may form PS‐SeNPs with SeNPs mainly through hydrogen bonding between Se^**…**^O–H and Se^**…**^N–H. CMC9, CMC25, and SeNPs mainly form PS‐SeNPs through hydrogen bonding between Se^**…**^ O–H.

The unique physicochemical properties established above, particularly the changes in surface charge and particle size, may determine the interaction between PS‐SeNPs and the gut microbiota. Given that bacterial cell envelopes typically carry a net negative charge, the cationic nature of CS‐Se, HACC‐Se, and COS‐Se suggests a potential for enhanced electrostatic attraction and adhesion to bacterial surfaces. In contrast, the anionic and larger‐sized CMC‐Se variants may experience electrostatic repulsion. Furthermore, the superior hydrophilicity and smaller size of COS‐Se could facilitate better dispersion and cellular contact in the aqueous fermentation environment. To investigate how these surface characteristics influence biological fate and metabolic modulation, we subsequently evaluated the in vitro fermentation performance.

### 3.2 Changes in pH Value

**FIGURE 2:**
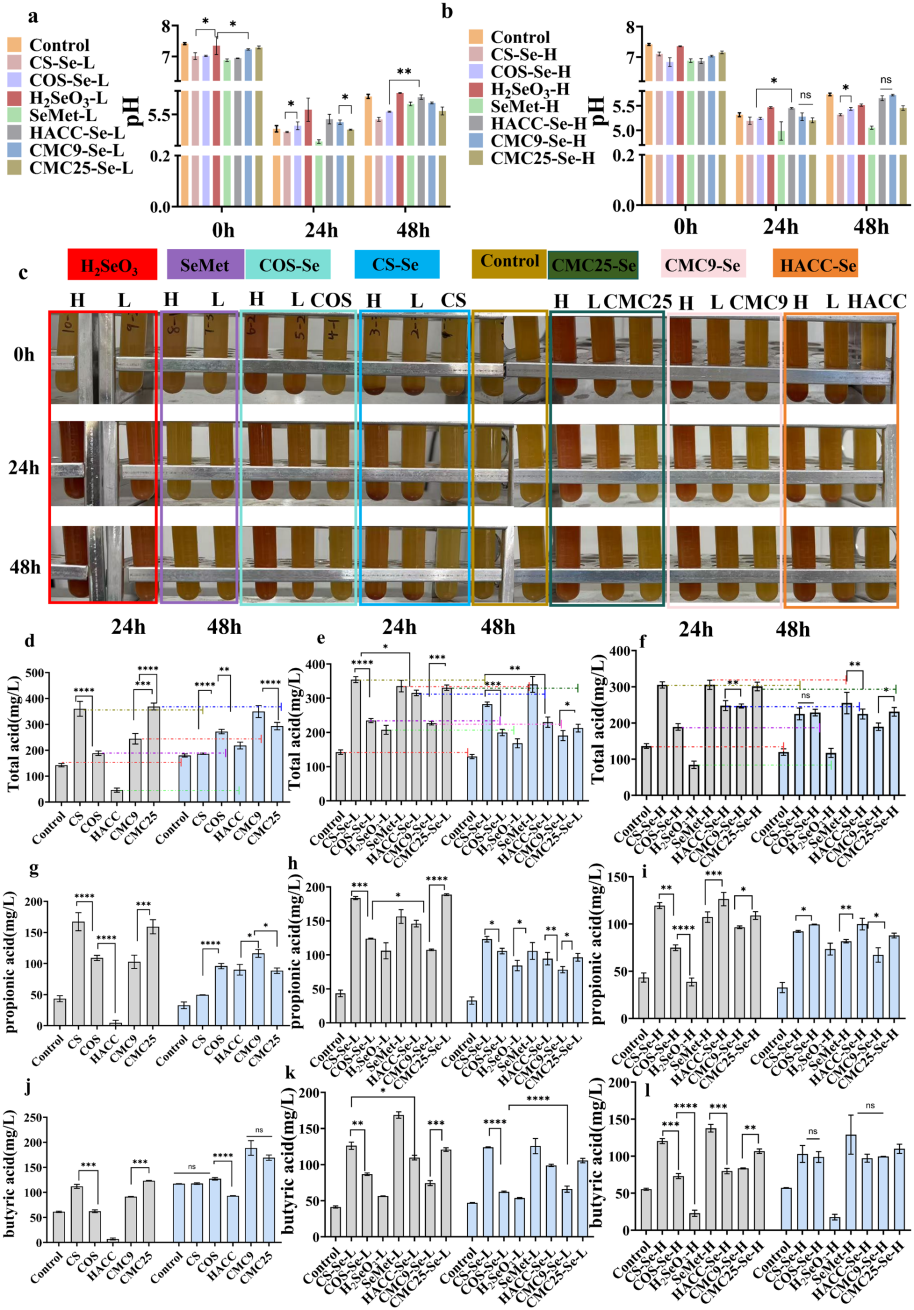
pH values of (a) PS‐SeNPs‐L and (b) PS‐SeNPs‐H after in vitro fermentation. (c) Color changes of different fecal fermentation samples at different fermentation times (H is high concentration, L is low concentration). The content of total acid produced by PS (d), PS‐SeNPs‐L (e), and PS‐SeNPs‐H (f) at 24 and 48 h. The content of propionic acid produced by PS (g), PS‐SeNPs‐L (h), and PS‐SeNPs‐H (i) at 24 and 48 h. The content of butyric acid produced by PS (j), PS‐SeNPs‐L (k), and PS‐SeNPs‐H (l) at 24 and 48 h. * *p* < 0.05, ** *p* < 0.01, *** *p* < 0.001, **** *p* < 0.0001.

The pH values of each PS group did not differ significantly, with the lowest pH value observed at 24 h (Figure S1a). The pH values of each PS‐SeNPs group decreased significantly compared with the control group (Figure 2a,b), indicating that more SCFAs were likely produced. Liang et al. studied the effects of different Se compounds on fecal microbiota. Their results indicate that, compared to the control group, all Se compounds cause a decrease in the pH value of the fermentation broth, which is consistent with the findings of this study (Liang et al. 2024).

### 3.3 Gut Microbiota Synthesizes Red SeNPs

Over time (0–48 h), the fermentation broth exposed to selenite showed a significant color change from yellow to red, indicating the in situ synthesis of SeNPs. Typically, the reduction of selenite (SeO_3_^2−^) to elemental Se (Se^0^) by the gut microbiota serves as a protective mechanism to mitigate Se toxicity, which is frequently observed as a characteristic red hue due to the formation of Se^0^ nanostructures. As fermentation progresses, the number and size of the particles increase or they aggregate, causing the color changes from red to dark red and brick red (Figure 2c). The color change indicates that selenite must undergo microbial reduction metabolism to be converted into a form that can be utilized by the host. The intermediate products generated during this process may be toxic to certain microbial communities, inhibiting their growth. This finding is consistent with previous research results (Liang et al. 2024). Additionally, under low concentration conditions, all PS‐SeNPs except for CMC25‐Se and CMC9‐Se did not exhibit significant color changes during fermentation, indicating higher biological safety. However, under high concentration conditions, except for CS‐Se, the remaining PS‐SeNPs exhibit noticeable color changes, indicating that some PS struggle to fully maintain the stability of SeNPs in this context. The presence of SeO_3_^2−^ in the solution requires microbial reduction to convert it into the less toxic Se ^0^. It is particularly noteworthy that CS‐Se did not exhibit significant color changes under low and high concentration conditions, indicating that CS‐Se has low biotoxicity, high physical and chemical stability, and good biocompatibility. Similarly, no significant color changes were observed in the gut microbiota treated with SeMet.

### 3.4 SCFAs

**FIGURE 3:**
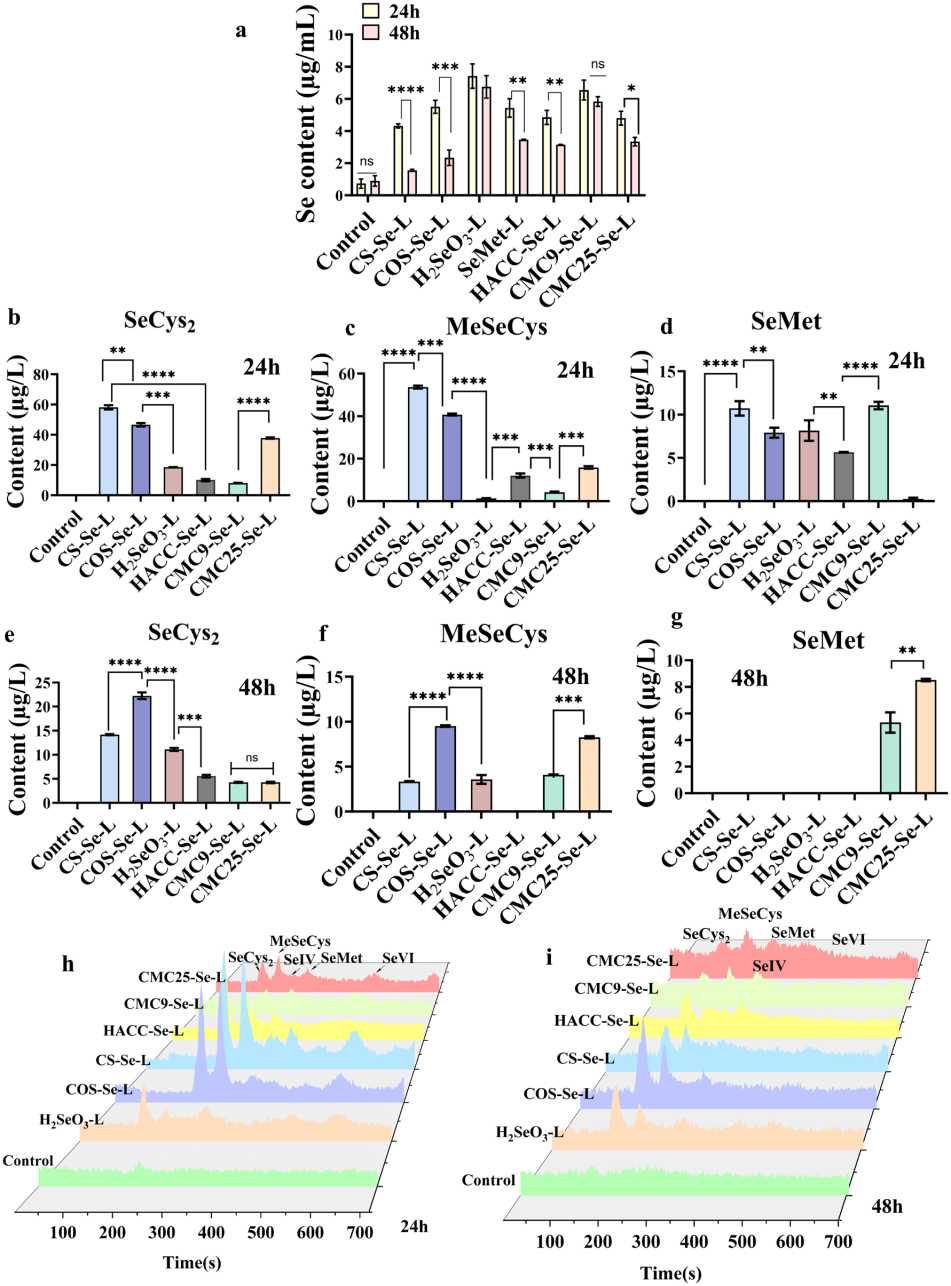
(a) The content of total Se (24 h and 48 h). (b) The content of SeCys_2_ (24 h). (c) The content of MetSeCys (24 h). (d) The content of SeMet (24 h). (e) The content of SeCys_2_ (48 h). (f) The content of MetSeCys (48 h). (g) The content of SeMet (48 h). The Se forms in different PS‐SeNPs fermentation solutions. (h) 24 h and (i) 48 h. * *p* < 0.05, ** *p* < 0.01, *** *p* < 0.001, **** *p* < 0.0001.

Figure 2d–l illustrates the changes in total acid, butyric acid, and propionic acid during 24 and 48 h of in vitro fermentation influenced by Se compounds. Figure S1b–d illustrates the changes in acetic acid during 24 and 48 h of in vitro fermentation influenced by Se compounds. Except for H_2_SeO_3_, the pH values in all Se compound treatments were generally lower than those in the control group, and the total SCFAs concentration was higher than that in the control group. After 24 h of fermentation with PS‐SeNPs‐L, the total SCFA content was as follows: H_2_SeO_3_ = CMC9‐Se < COS‐Se < HACC‐Se < SeMet = CMC25‐Se < CS‐Se (Figure 2e). As the fermentation time extended to 48 h, the total content of SCFAs showed a significant decline compared to 24 h (24 h vs. 48 h; Figure 2e,f). In addition, a significant decrease in selenium content was observed during the later stages of fermentation (Figure 3a), consistent with the aforementioned changes. In the early stages, active microbial metabolism may promote the adsorption, transformation, or integration of SeNPs. However, as readily available selenium sources become depleted and microbial activity diminishes due to complex substrates, the bioavailability and fixation efficiency of selenium also decrease, leading to a reduction in SCFAs (24 h vs. 48 h; Figure 2e,f). In addition to total SCFAs, the concentrations of major SCFAs such as acetic acid, propionic acid, and butyric acid followed the same dynamic pattern, with levels at 48 h of fermentation being lower than those at 24 h (24 h vs. 48 h; Figures 2g–l and S1b–d). The results showed that the promoting effect of PS‐SeNPs on the production of SCFAs exhibited a non‐linear dose‐dependent relationship: the treatment effect was significant at low concentration (PS‐SeNPs‐L), while increasing the concentration (PS‐SeNPs‐H) did not further enhance this effect (low concentration vs high concentration) (Figure 2e,f).

Compared to organic Se compounds, the fermentation efficiency of H_2_SeO_3_ is relatively low. This difference may be attributed to microorganisms preferentially utilizing less toxic organic Se rather than inorganic selenite (Zhang et al. 2020). However, regarding SCFA production, the effectiveness of SeNPs stabilized by low molecular weight PS (COS‐Se and CMC9‐Se) was lower than that of CMC25‐Se and CS‐Se. Additionally, after 24 h of fermentation, CMC9‐Se exhibited the lowest SCFA production, while CS‐Se demonstrated the highest SCFA production. According to existing research reports (Yu et al. 2012), this difference may be related to the surface charge of nanoparticles; specifically, positively charged nanoparticles are more likely to adsorb onto negatively charged bacterial surfaces, thereby enhancing bacterial uptake. Compared to low molecular weight PS, previous studies have shown that SeNPs stabilized with high molecular weight PS not only release Se more readily but are also absorbed more efficiently due to electrostatic effects (Song, Chen, Zhao, et al. 2020). This mechanism requires further validation through subsequent experiments in this study. Further analysis revealed that HACC in vitro fermentation significantly inhibited SCFAs production (Figure 2d,g), consistent with results reported in the literature (Cheah et al. 2019; Jiao et al. 2017; Liu et al. 2025), whereas HACC‐stabilized SeNPs (HACC‐Se) significantly enhanced SCFAs levels (Figure 2e,f), revealing complex interactions between PS‐SeNPs and microbial metabolism. However, when HACC binds to SeNPs as a skeleton to form HACC‐Se, its interaction with the microbial community undergoes fundamental changes. This transformation may be attributed to the formation of nanostructures. Specifically, during the synthesis of SeNPs, HACC interacts with selenium primarily through Se… O–H and Se… N–H hydrogen bonds to form PS‐SeNPs (Figure 1f). At the same time, the conformation of HACC changed from a linear form to circular particles (HACC‐Se), thereby altering its bioavailability to microorganisms (Figure 1a) (Dai et al. 2025). Finally, the selenium released by SeNPs can enhance the antioxidant stress resistance of bacteria (e.g., by increasing selenoprotein synthesis), which may, to some extent, counteract the stress response induced by HACC (Rayman 2012). Therefore, the promoting effect of HACC‐Se on SCFAs is not due to the polysaccharide carrier alone, but rather the combined synergistic effect of the stable SeNPs core and the unique physicochemical properties of HACC. The molecular characteristics of PS‐including their functional groups, surface charge, and molecular weight‐collectively create a multidimensional regulatory system that governs the interaction patterns and biological effects between SeNPs and gut microbiota.

We comprehensively evaluated efficiency and cost, establishing PS‐SeNPs‐L as the primary research focus due to its dual advantages in promoting SCFA production (Figure 2d–l) and biocompatibility (Figure 2c). Regarding time parameters, since SCFA levels at 48 h of fermentation were lower than at 24 h—indicating metabolic consumption during the later fermentation stage—we ultimately selected 24 h as the optimal time window to capture the peak metabolic characteristics.

### 3.5 Se Form

Many studies have emphasized the ability of gut microbiota to absorb Se, thereby enhancing its metabolic processes (Chen et al. 2025; Pophaly et al. 2014; Zhang et al. 2025). To investigate the effect of gut microbiota on the bioavailability of Se compounds. This study analyzed selenium forms in the fermentation supernatant. ICP‐MS analysis confirmed that the initial selenium concentration across PS‐SeNPs‐L groups was maintained at approximately 10 μg/mL, with no significant differences observed (*p* > 0.05). The Se content gradually decreased during the 24 to 48 h, indicating that Se compounds are assimilated by the gut microbiota (Figure 3a) (Liang et al. 2024; Takahashi et al. 2020). This is consistent with the changes in SCFAs (Figure 2). Compared to organic Se compounds, H_2_SeO_3_ showed a lower rate of microbial assimilation (Jäger et al. 2016; Shi et al. 2021), with the Se content decreasing by only 0.67 μg/mL during the 24 h fermentation period. The absorption efficiency of organic Se is significantly higher than that of H_2_SeO_3_, indicating the presence of a preferential assimilation mechanism within the gut microbiota (Liang et al. 2024; Zhao et al. 2021). H_2_SeO_3_ must undergo an energy‐consuming reduction process before it can be utilized by microorganisms. These microorganisms consume their own NADPH and ATP to gradually reduce H_2_SeO_3_ to the intermediate H_2_Se, activate selenophosphate (SePO_3_^3−^) and selenocysteine tRNA^[Ser]Sec^ (Sec tRNA), and regulate the production of selenoproteins (Zhao et al. 2021). The essence of the “priority assimilation mechanism” is a strategy developed by microorganisms through long‐term evolution, aimed at maximizing metabolic efficiency while minimizing survival risks.

After 24 h of fermentation, the Se (IV) concentration in all inorganic selenium groups was zero, indicating that the inorganic selenium source had been completely reduced by the microorganisms. Regarding total selenium consumption, the CS‐Se group demonstrated the highest conversion efficiency, with a reduction of 2.763 μg/mL in selenium content—significantly greater than the reductions observed in the HACC‐Se (1.70 μg/mL), CMC9‐Se (0.71 μg/mL), and CMC25‐Se (1.460 μg/mL) group (Figure 3a). The main conversion products of SeNPs were identified as SeCys_2_, SeMet, and MeSeCys (Figure 3h,i). Similarly, different PS‐SeNPs exhibited significant differences in transformation, highlighting the crucial role of the carrier structure. CS‐Se and COS‐Se demonstrated efficient conversion of SeCys_2_ at concentrations of 58.13 and 46.6 μg/L, MeSeCys at 53.61 and 40.71 μg/L, SeMet at 10.71 and 7.91 μg/L after 24 h of fermentation, which were significantly higher than those in the H_2_SeO_3_ group (18.52, 1.37 and 8.16 μg/L, respectively; Figure 3b–d). This enhanced transformation is speculated to be attributed to the positive charge characteristics of CS and COS, which may increase the adsorption and uptake capacity of bacterial cell membranes, thereby promoting microbial assimilation of selenium. Compared to the CS‐Se group, the conversion efficiency of the negatively charged CMC‐Se group is significantly lower. The above mechanism needs further experimental confirmation in this study. The SeCys_2_ and MeSeCys produced by the CMC9‐Se group were only 8.01 and 4.26 μg/L, respectively. Although the CMC25‐Se group showed a slight increase (SeCys_2_ at 37.8 μg/L), MeSeCys (16.87 μg/L) remained much lower than in the CS‐Se and COS‐Se groups (Figure 3b–d,h). As fermentation progressed to 48 h, the selenium concentration showed a significant downward trend. For example, in the CS‐Se group, SeCys_2_ decreased from 58.13 to 14.17 μg/L, while MeSeCys dropped sharply from 53.61 to 3.34 μg/L (Figure 3e–g,i). This observation confirms that the organic selenium produced was preferentially absorbed and incorporated into bacterial proteins by the microbial community, rather than accumulating in the environment. In summary, the absorption and conversion efficiency of Se compounds by gut microbiota is highly dependent on the structural characteristics of PS (Shi et al. 2021). Compared to inorganic Se, organic Se and PS‐SeNPs are more readily assimilated and transformed by gut microbiota, highlighting the crucial role of PS in regulating Se microbial metabolism.

### 3.6 Effects of in vitro Fermentation of Se Compound Protein on Gut Microbiota

#### 3.6.1 Alpha and Beta Diversity Analysis

**FIGURE 4:**
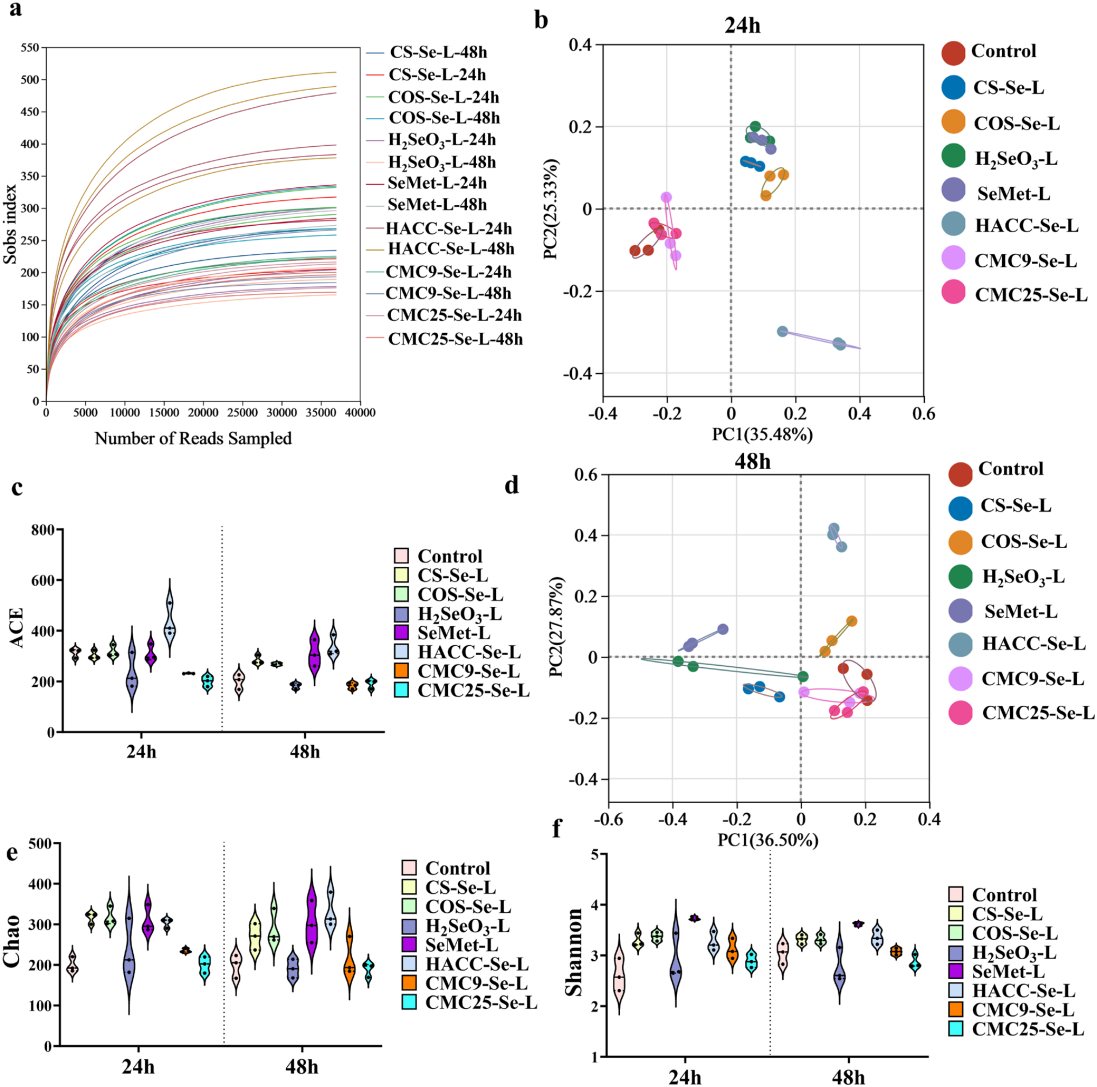
(a) Rarefaction curve. (b) Principal component analysis of low‐concentration PS‐SeNPs in 24 h. (c) ACE index. (d) Principal component analysis of low‐concentration PS‐SeNPs in 48 h. (e) Chao index. (f) Shannon index.

To further elucidate the changes in the gut microbiota induced by distinct chemical forms of Se and PS‐SeNPs, this study employed an in vitro fermentation model to assess the differential effects on gut microbiota composition and structure. As shown in Figure 4a, the curve of rarefaction arrived at a stable point with the increase of sequencing depth, indicating that the sequencing depth has captured the vast majority of microbial information and is sufficient for subsequent analysis. The results showed that the richness and diversity of microbial communities in each experimental group increased compared to the control group (Figure 4c,e,f), suggesting that all kinds of Se may have the potential to improve microbial community structure (Wang, Wang, et al. 2025). As shown in Figure 4b, except for the CMC9‐Se and CMC25‐Se groups, all other experimental groups were significantly separated from the control group, indicating a substantial change in their microbial composition. Meanwhile, partial overlap was observed between the CMC9‐Se and CMC25‐Se groups, suggesting that the gut microbiota structures of these two groups were relatively similar. Based on the preceding analysis of Se utilization efficiency, the question arises as to whether CMC9‐Se and CMC25‐Se have no influence on the structure and metabolism of the microbiota. To address this, we conducted a further analysis of the composition and relative abundance of gut microbiota.

#### 3.6.2 Composition and Relative Abundance of Gut Microbiota

**FIGURE 5:**
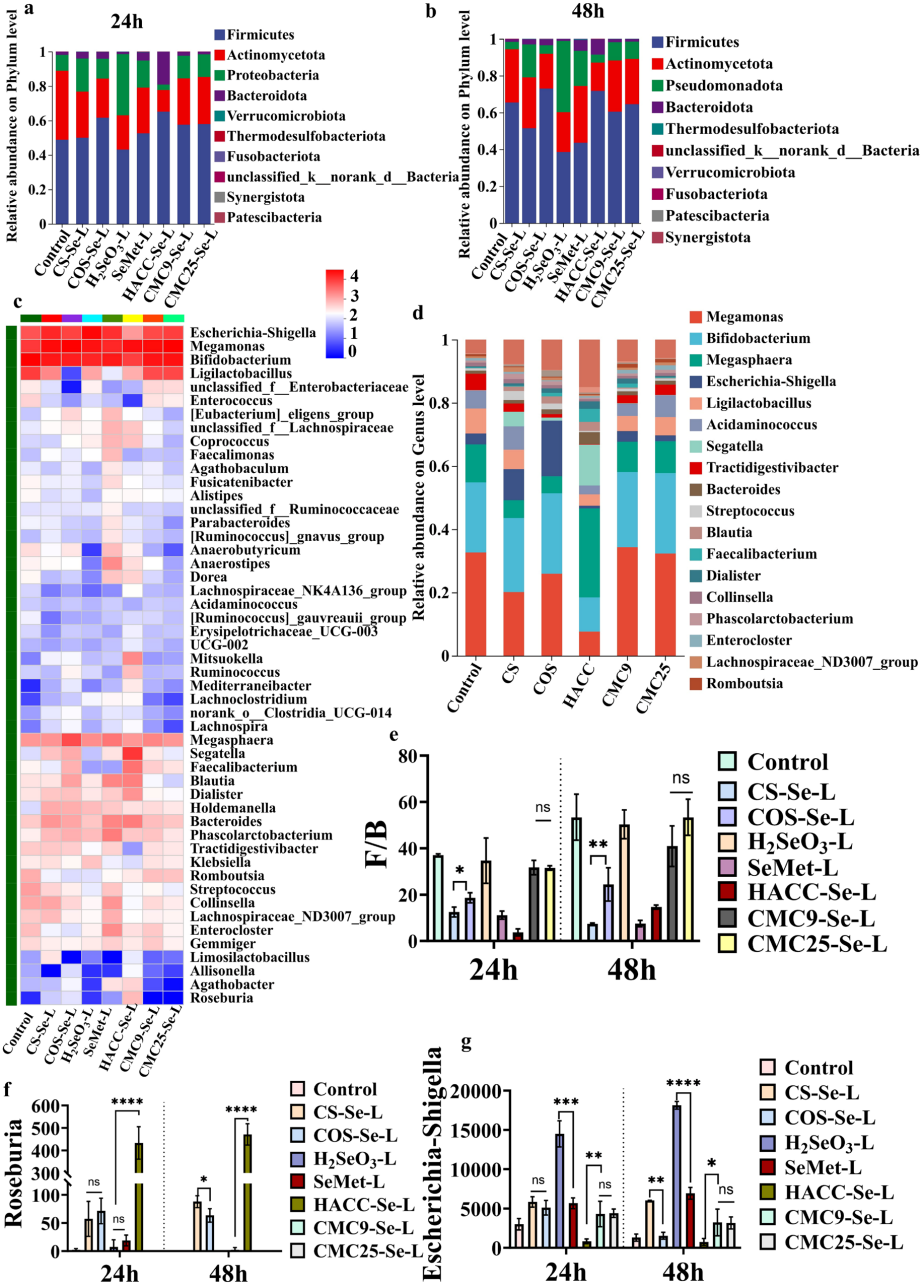
(a) Barplot chart of relative abundances of gut microbiota at the phylum level of PS‐SeNPs‐L after 24 h of fermentation. (b) Barplot chart of relative abundances of gut microbiota at the phylum level of PS‐SeNPs‐L after 48 h of fermentation. (c) Heat map of relative abundances of gut microbiota at the genus level of PS‐SeNPs‐L after 24 h of fermentation. (d) Barplot chart of relative abundances of gut microbiota at the genus level of PS after 24 h of fermentation. (e) The ratio of *Firmcutes/Bacteroidota*. The relative abundance of (f) *Roseburia*, (g) *Escherichia‐Shigella*. * *p* < 0.05, ** *p* < 0.01, *** *p* < 0.001, **** *p* < 0.0001.

After 24 h, at the phylum level, we identified four dominant bacteria, namely *Firmicutes, Actinomycetota, Proteobacteria*, and *Bacteroidota* (Figure 5a). With the increase of time, the abundance of *Firmicutes* increased. Compared to the SeMet group, the H_2_SeO_3_ treatment group significantly decreased the relative abundance of *Bacteroidota* and *Proteobacteria*, leading to a significant increase in the *Firmicutes/Bacteroidota* (F/B) ratio (Figure 5a,b,e). Previous studies have shown that an increase in F/B ratio is closely associated with a higher risk of gut microbiota dysbiosis (Chang et al. 2015). Compared to the control group, all PS‐SeNPs treatment groups exhibited a trend toward reducing the F/B ratio, suggesting a potential restorative effect on microbial dysbiosis. The COS‐Se group significantly increased the relative abundance of *Firmicutes* at the phylum level. Therefore, compared with the CS‐Se group, it exhibited a higher F/B ratio; however, this ratio remained significantly lower than that of the H_2_SeO_3_ group and the control group (Figure 5e). The CS‐Se group exhibited a lower abundance of *Firmicutes* and reduced F/B ratios and promoted the growth of *Bacteroidota* (Figure 5a,b). According to existing research, the molecular weight of polysaccharides may play a significant role in this process (Chen et al. 2019; Song, Chen, Zhao, et al. 2020). It is speculated that the CS‐Se group performs better in maintaining microbial balance, which may be related to this factor. Compared with the other treatment groups, the F/B ratios in the CMC9‐Se and CMC25‐Se groups were higher but remained lower than those in the control group. From the perspective of charge characteristics, we speculate that SeNPs modified with positively charged polysaccharides may exhibit a stronger affinity for negatively charged bacterial cell membranes. This mechanism has been reported in the literature (Lee et al. 2024). In summary, PS‐SeNPs with varying molecular weights and surface characteristics exhibit significant regulatory effects on the composition of the gut microbiota.

At the genus level, the results of in vitro fermentation experiments showed that compared with the control group, HACC‐Se promotes the abundance of beneficial bacteria such as *Lachnospiraceae_NK4A136, Roseburia, Bacteroides, Ruminococcus*, and *Anaerostipes*, while inhibiting the proliferation of *Escherichia‐Shigella* and *Enterococcus* (Figure 5c,f,g). Compared with the other groups, the H_2_SeO_3_ group exhibited the highest abundance of *Klebsiella* and *Escherichia‐Shigella* (Figure 5c,g), suggesting that under simulated colonic conditions, inorganic Se may have a limited capacity to modulate the gut microbiota. Compared to the control group, the abundance of *Segatella, Faecalimonas, Lachnospiraceae_ND3007 group, Roseburia*, *Eubacterium_Eligens*, and *Limosilactobacillus* was significantly increased in the CS‐Se group (Figure 5c,d,f). In the COS‐Se group, *Roseburia, Eubacterium_Eligens*, and *Holdemanella* showed significant changes (Figure 5c). The CMC9‐Se and CMC25‐Se groups exhibited similar trends in modulating gut microbiota composition. Both CMC9‐Se and CMC25‐Se had limited effects in inhibiting potentially harmful bacteria; however, they promoted the proliferation of certain probiotic genera, such as *Ligilactobacillus* and *Faecalibacterium* (Figure 5c). The findings regarding these SCFAs and the Se form are consistent.

**FIGURE 6:**
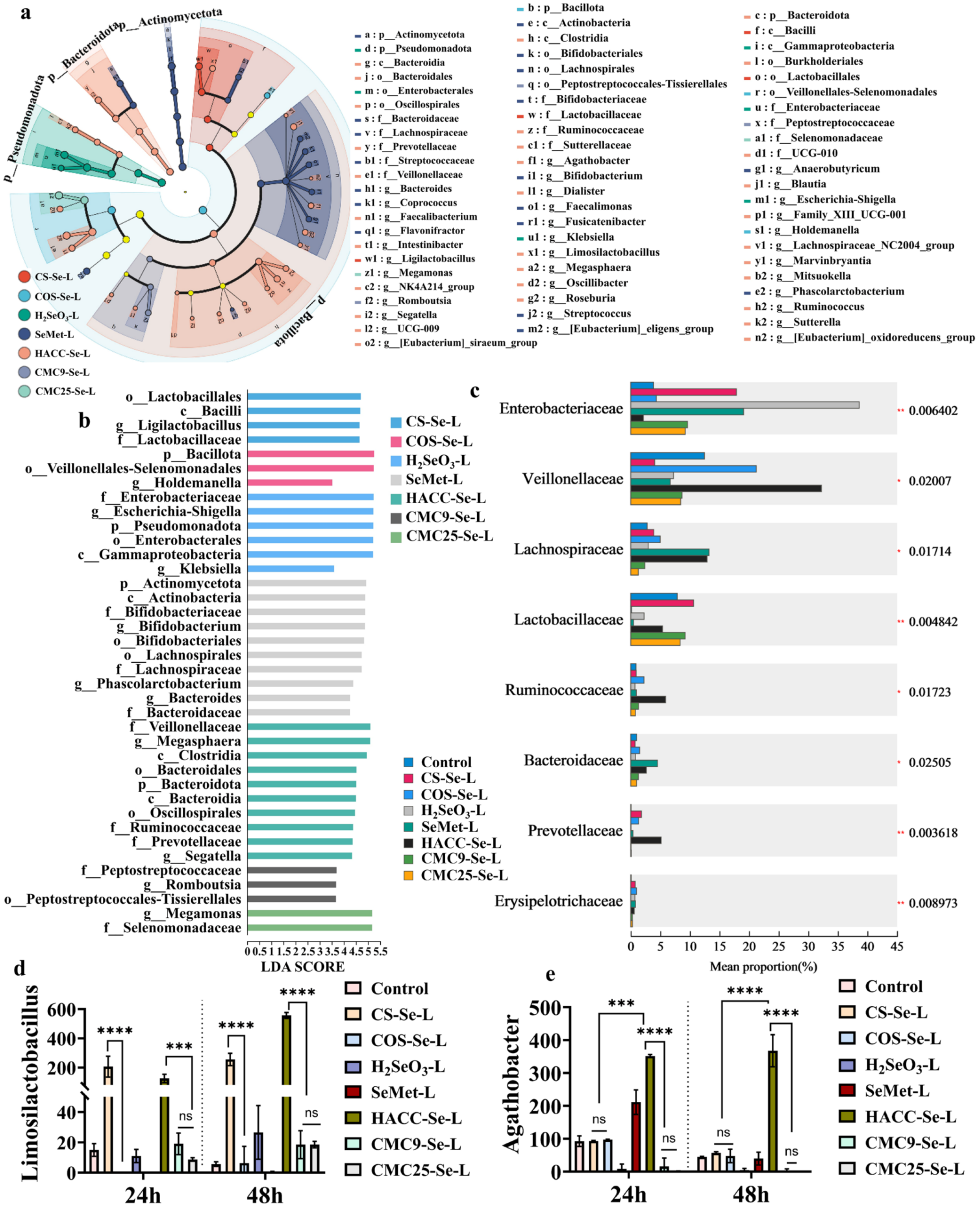
(a) LEfSe multi‐level species hierarchical tree diagram and (b) LDA scores. (c) The significant differences of the same species among multiple different groups. The relative abundance of (d) *Limosilactobacillus* and (e) *Agathobacter*. * *p* < 0.05, ** *p* < 0.01, *** *p* < 0.001, **** *p* < 0.0001.

Meanwhile, we used Linear Discriminant Analysis Effect Size (LEfSe) and LDA scores to illustrate the differences in gut microbiota profiles among different treatment groups (Figure 6a,b). CS‐Se significantly enriched core microbial communities capable of polysaccharide degradation and acid production, including *Lactobacillales, Lactobacillaceae*, and *Ligilactobacillus* (Figure 6a,b). These microbial communities not only promoted the accumulation of SCFAs such as propionic acid and butyric acid (Figure 2), but were also deeply involved in selenium metabolism. Correspondingly, the yields of SCFAs, SeCys_2_, and MeSeCys in the CS‐Se group were significantly higher than those in the other treatment groups (Figure 3b–d), which was significantly positively correlated with the abundance of genera such as *Lactobacillaceae* in this group. This finding is consistent with the results of Eliza Kurek and M. Calomme et al. *Lactobacillaceae* participate in Se metabolism, producing SeCys_2_, MeSeCys, and other undetermined forms (Calomme et al. 1995). The COS‐Se group primarily enriched *Bacillota, Velionellales‐Selenomonadales*, and *Holdemanella* (Figure 6b); however, their total SCFA and selenium metabolite levels were lower than those of the CS‐Se group (Figures 2 and 3). Combining differences in molecular weight, high‐molecular‐weight polysaccharides may be more effective in enriching functional microbial communities capable of selenium conversion and amino acid synthesis, thereby enhancing overall metabolic efficiency. SeMet can effectively promote the growth of beneficial bacteria such as *Bifidobacterium*, *Bacteroides*, and *Phascolarctobacterium*, which possess strong Se assimilation and SCFAs generation abilities. For CMC9‐Se and CMC25‐Se, their inhibitory effects on potentially harmful bacteria are limited; however, they also promote the proliferation of certain beneficial genera, such as *Romboutsia* and *Lactobacillaceae* (Figure 6b,c). According to literature reports, it is speculated that negative surface charges may weaken the electrostatic adsorption of bacterial cell membranes, thereby reducing the absorption and conversion efficiency of selenium by functional microbial communities (Yu et al. 2012). It is worth noting that, compared with CMC9‐Se, CMC25‐Se further increased the relative abundance of *Selenomonadaceae*, indicating that even subtle differences in molecular weight can affect the microbial response to negatively charged SeNPs. The HACC‐Se group exhibited a unique regulatory pattern. HACC alone inhibited microbial growth and SCFAs production, and reduced the abundance of *Bifidobacterium* and *Ligilactobacillus* (Figure 5d); HACC‐Se significantly enriched beneficial bacterial communities such as *Bacteroidales, Ruminococcaceceae*, *Agathobacter* and *Prevotellaceae*, while inhibiting pathogenic bacteria such as *Shigella* (Figure 6b,c,e). Although the level of selenium metabolites in this group was lower than that in the CS‐Se group, its SCFA production was significantly higher than that in the HACC group and most other treatment groups (Figure 2). This indicates that the regulatory effect of this system on microbial metabolism may result from the synergistic interaction between SeNPs and the physicochemical properties of the polysaccharide carrier. Simultaneously, they create a more favorable microbial environment for Se metabolism and transformation (Zhang et al. 2024). The H_2_SeO_3_ group showed an enrichment of opportunistic pathogens, such as *Enterobacteriaceae* and *Escherichia‐Shigella* (Figures 5g and 6b), while the abundance of probiotic groups decreased, leading to a reduced level of SCFAs production (Figure 2).

Overall, CS‐Se can significantly enrich probiotics (such as *Lactobacillaceae*) with both acid production and Se metabolism capabilities, thereby creating a beneficial cycle of efficient organic Se conversion and increased SCFAs production. In contrast, the probiotic effects of low molecular weight polysaccharide systems (COS‐Se) or negatively charged systems (CMC‐Se) are weaker, resulting in reduced levels of SCFAs and lower Se bioavailability. It is worth noting that HACC inhibits microbial growth and metabolism to some extent, as evidenced by reduced levels of *Bifidobacterium, Megamonas, Ligilactobacillus*, and SCFAs (Figures 3 and 5d; Pophaly et al. 2014). However, in the HACC‐Se group, changes in surface physicochemical properties or the provision of specific substrate structures guide the microbial community to shift significantly toward beneficial metabolic activity, demonstrating effective regulation of the microbial community. In contrast, H_2_SeO_3_ tends to promote the proliferation of pathogenic bacteria such as *Enterobacteriaceae* (Figure 6c), while inhibiting beneficial bacterial communities and their functions, which adversely affects the benign transformation of Se and metabolic homeostasis.

### 3.7 Metabolomics Analysis

**FIGURE 7:**
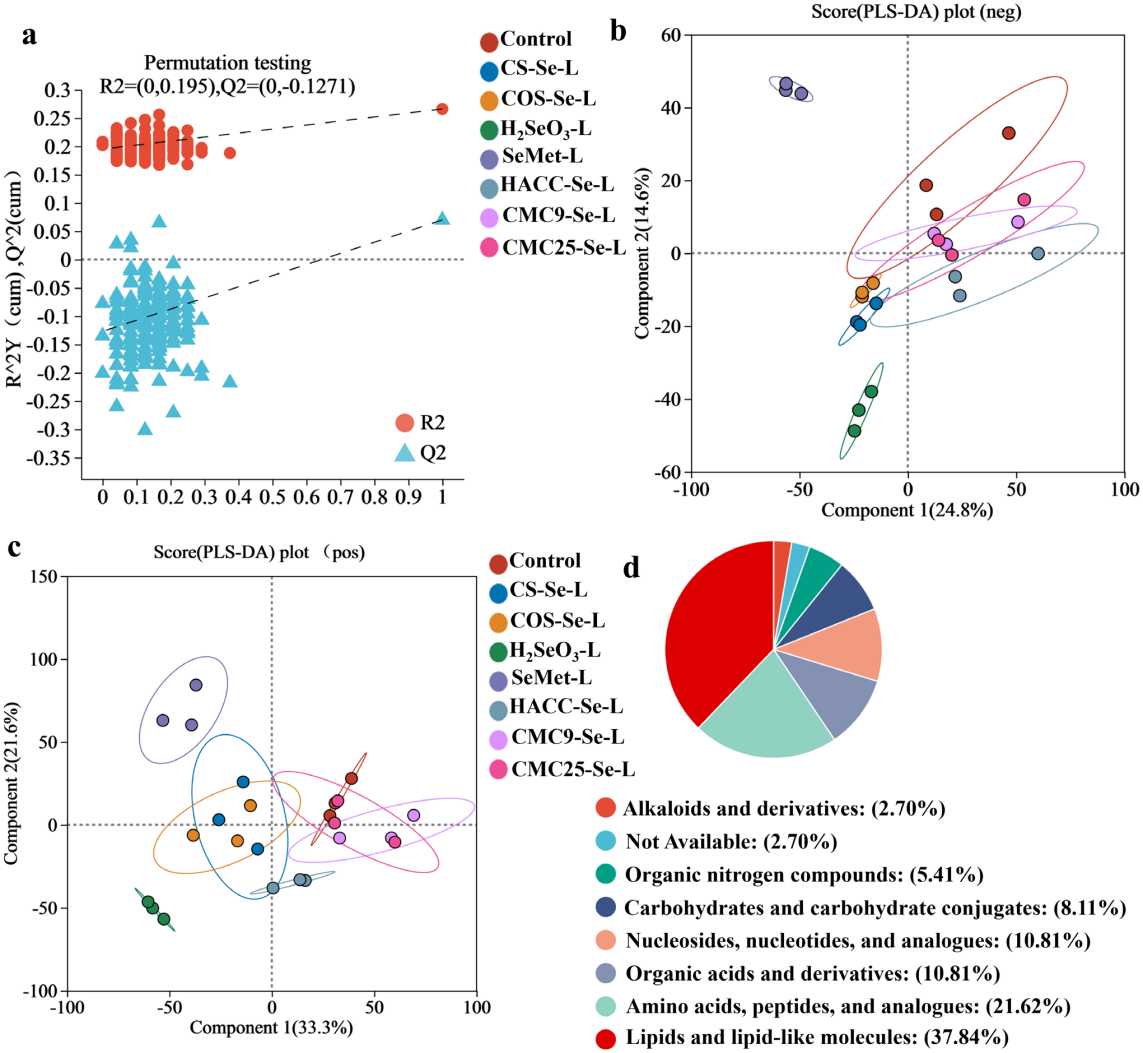
(a) PLS‐DA score plot. (b) Principal component analysis (NEG, negative ion mode). (c) Principal component analysis (POS, positive ion mode). (d) Percentage of identified intracellular metabolites.

To further analyze the regulatory effects of different PS‐SeNPs on gut microbiota metabolic function. By identifying differential metabolites and enriching metabolic pathways, we aim to elucidate the metabolic differences caused by different PS‐SeNPs. The validity of the PLS‐DA model was confirmed through permutation testing, and it was determined that the model did not overfit (Figure 7a). As shown in Figure 7c,d, the SeMet and H_2_SeO_3_ groups were significantly separated from the other sample points, indicating a substantial change in the composition of their metabolites. However, there is partial overlap in the distribution between different PS‐SeNPs groups, indicating that the metabolic pathways of these groups may have certain commonalities. We use the Human Metabolome Database (HMDB) to classify metabolites, mainly including Lipids and lipid‐like molecules, Amino acids, peptides, and analogues, Organic acids and derivatives, Nucleosides, nucleotides, and analogues, Carbohydrates and carbohydrate conjugates (Figure 7d).

**FIGURE 8:**
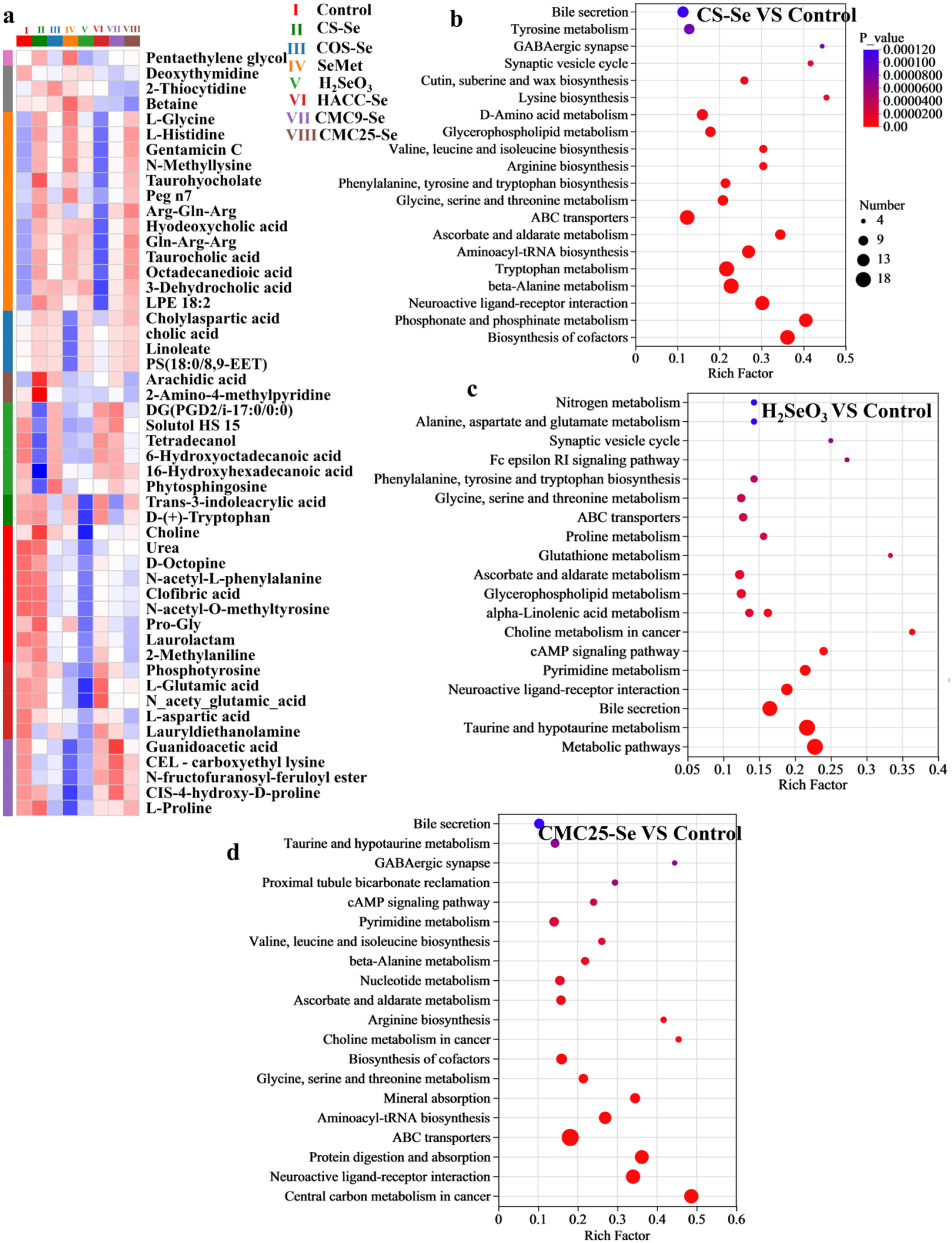
(a) Heatmap alignment of differential metabolites in different PS‐SeNPs. (b–d) Analysis of KEGG pathway enrichment in different comparative treatment groups.

Through the analysis of metabolite heatmaps, the differential metabolites between different experimental groups are mainly divided into the following categories. The first type of metabolites includes amino acids, dipeptides, and acetylated amino acids such as L‐Glycine, L‐Histidine, acid, Trans‐3‐indoleacrylic acid, D‐(+)‐Tryptophan, CIS‐4‐hydroxy‐D‐proline, L‐Proline. The second type of metabolites is lipids, metabolites of steroids: 10‐Heptadecenoic acid, Linoleate, Arachidonic acid, LPE 18:2, LPE 18:1, PS (18:0/8,9‐EET), etc. The third type of nucleotide metabolite is a small amount of carbohydrate metabolite: 2‐Thiocytidine, Deoxythymidine, Glucaric acid, etc. Amino acids and their metabolites showed a significant downregulation trend in the H_2_SeO_3_ group. The content of various amino acids and their derivatives, such as L‐Glycine, L‐Histidine, L‐Glutamic acid, D‐(+)‐Tryptophan, and various acetylated amino acids such as N‐acetyl‐L‐phenylalanine, has decreased (Figure 8a). This suggests that H_2_SeO_3_ may inhibit the microbial synthesis of Nitrogen metabolism, Alanine, aspartate and glutamate metabolism, Phenylalanine, tyrosine and tryptophan biosynthesis, Glycine, serine and threonine metabolism, ABC transporters, Glutathione metabolism, Ascorbate and aldarate metabolism, Glycerophospholipid metabolism, alpha‐Linolenic acid metabolism, Choline metabolism in cancer, cAMP signaling pathway, Taurine and hypotaurine metabolism, etc. (Figure 8c). The metabolic pathways significantly altered in the CS‐Se group include Lysine biosynthesis, D‐Amino acid metabolism, Glycerophospholipid metabolism, Valine, leucine and isoleucine biosynthesis, Arginine biosynthesis, Phenylalanine, tyrosine and tryptophan biosynthesis, Glycine, serine and threonine metabolism, ABC transporters, Ascorbate and aldarate metabolism, Aminoacyl‐tRNA biosynthesis, Tryptophan metabolism, beta‐Alanine metabolism, Neuroactive ligand‐receptor interaction, Phosphonate and phosphinate metabolism, etc. (Figure 8b). Figures 8d and S2a–c respectively show the metabolic pathway differences between CMC25‐Se, COS‐Se, HACC‐Se, and CMC9‐Se compared to control. COS‐Se, CMC9‐Se, CMC25‐Se, and HACC‐Se also significantly altered the metabolic pathways of certain amino acids, organismal Systems, human diseases and fatty acids, consistent with previous literature reports (Parra‐Martínez et al. 2022; Yang et al. 2025). It is worth noting that there are significant differences in the regulatory effects of SeNPs formed by PS with different structures on amino acid and fatty acid metabolism pathways. Taking the Lysine biosynthesis pathway as an example, the CS‐Se group significantly influenced this pathway (Figure 8b). Figure S3b shows the metabolite differences between CS‐Se and COS‐Se. In this metabolic pathway, 2‐oxoglutaric acid is metabolized to L‐lysine, after which 2‐oxoglutaric acid and L‐lysine are condensed to form L‐saccharopine (Xu et al. 2006; Figure S3b). The results showed that the levels of 2‐oxoglutaric acid and L‐lysine in the CS‐Se group exhibited an upregulation trend, whereas in the COS‐Se group, the levels of these two metabolites decreased significantly. This indicates that the regulation of lysine metabolism by different PS‐SeNPs is structurally dependent. Combined with microbiome analysis, CS‐Se significantly enriched *Lactobacillaceae* and *Lactobacillus*, which possess strong amino acid synthesis capabilities, whereas COS‐Se enriched bacteria such as *Romboutsia*, which have comparatively weaker amino acid synthesis abilities. In addition, the elevated levels of SCFAs induced by CS‐Se can enter the tricarboxylic acid cycle, supplying essential carbon skeleton precursors for lysine biosynthesis. Notably, compared to other PS‐SeNPs (particularly CMC25‐Se), all treatment groups exhibited distinct changes in the phenylalanine, aspartic acid, and glutamic acid metabolic pathways, with the CMC25‐Se group showing a significant reduction in L‐aspartic acid levels. It is a key node in this metabolic network, can directly promote the production of oxaloacetic acid through transamination. Oxaloacetic acid is believed to possess anti‐inflammatory properties and enhance glucose utilization by nerve cells (Wang et al. 2024). Figure S3a shows the metabolite differences between HACC‐Se and CMC9‐Se. The HACC‐Se group promoted the accumulation of amino acids, including Tyrosine, L‐serine, and L‐glutamic acid, whereas the levels of L‐glutamic acid decreased in the CMC9‐Se group (Figures 8a and S3a). CMC25‐Se and CMC9‐Se jointly cause an increase in L‐histidine and L‐Glycine levels (Figures 8a and S3a). These results further confirm the differential impact of PS structural characteristics on the metabolism of SeNPs.

After adding H_2_SeO_3_, the metabolism of some amino acids is more significantly inhibited (Guo and Jia 2025). Correspondingly, the H_2_SeO_3_ group exhibited a significant enrichment of opportunistic pathogens, such as *Escherichia‐Shigella*, along with the lowest SCFA production (Figure 2). In the process of arginine synthesis, L‐citrulline is sequentially catalyzed by argininosuccinate synthase 1 (ASS1) and argininosuccinate lyase (ASL) to produce L‐arginine, which can then be hydrolyzed by arginase to produce urea and L‐ornithine, thereby completing a partial urea cycle (Canè et al. 2025; Guo and Jia 2025). The urea level in this group decreased to its lowest point, indicating that arginine biosynthesis and the urea cycle were impaired (Figure 8a,c). Previous studies have reported that low doses of selenite (2 μg/g) do not significantly inhibit amino acid metabolism and synthesis processes; however, increasing Se concentration may inhibit ASS1 expression, which in turn disrupts arginine metabolism and synthesis homeostasis (Tian et al. 2022; Wang, Wang, et al. 2025). The high concentration of H_2_SeO_3_ used in this study may hinder arginine biosynthesis by inhibiting ASS1 enzyme activity. The L‐proline levels in the PS‐SeNPs group were significantly higher than those in the H_2_SeO_3_ group. Additionally, the L‐glutamate levels in the CS‐Se and HACC‐Se groups were significantly higher than those in the other PS‐SeNPs groups (Figures 8a,b and S2b). Both L‐glutamic acid and L‐proline play central roles in the central nervous system as key neurotransmitters (Dogra et al. 2022; Yao and Han 2022). The results of the metabolic pathway analysis showed that glutathione metabolism was significantly enriched in the H_2_SeO_3_‐treated group (Figure 8b). Under normal physiological conditions, glutathione can undergo detoxification reactions with selenite to produce selenodiglutathione, a key intermediate in the metabolic transformation of Se in the body (Lin et al. 2024; Roman et al. 2014). Se supplementation also particularly affects ABC transporter (Figures 8a–c and S2). This significant metabolic difference indicates that different Se not only affects the bioavailability of Se, but also reshapes the metabolic function of gut microbiota, which may have vastly different effects on host metabolism.

## 4 Conclusion

This study analyzed the complex interactions between PS‐SeNPs synthesized from various PS and microbial metabolism using an in vitro colon fermentation system. The results demonstrated that the molecular characteristics of PS significantly influence the bioavailability, microbial community composition, and metabolic pathways of Se. Research has found that CS‐Se and HACC‐Se significantly promote the accumulation of SCFAs and the transformation of biologically active Se forms by specifically enriching beneficial microbial communities, such as *Lactobacillus*, mainly promoting the biosynthesis and metabolic pathways of various amino acids. There were significant differences in the regulatory ability of COS‐Se, CMC9‐Se, and CMC25‐Se on gut microbiota composition and metabolic function, and the content of SCFAs produced is also lower than that of CS‐Se. The characteristics of PS are key factors in regulating the function of the gut microbiota and the metabolic activity of SeNPs. High molecular weight and positively charged PS enhance the microbial utilization of SeNPs. Additionally, low concentrations of PS‐SeNPs did not exhibit significant color changes during fermentation, suggesting better stability and lower microbial disturbance under the tested conditions. Compared to organic Se compounds, microorganisms show lower absorption and assimilation efficiency for H_2_SeO_3_, indicating that microorganisms may preferentially metabolize organic Se with higher bioavailability. Although this study systematically demonstrated the selective regulatory effects of PS‐SeNPs with different structures on gut microbiota metabolism using in vitro models, certain limitations remain. Due to significant individual differences in gut microbiota, the generalizability of these research findings to a broader population requires further validation. Future studies should increase the sample size and incorporate in vivo animal trials to comprehensively assess the probiotic effects of these selenium‐rich polysaccharide complexes within complex physiological environments. This study offers a valuable reference for the rational design of efficient and safe selenium nano‐supplements and underscores the potential of modulating gut microbiota through polysaccharide materials as a strategy for nutritional metabolism intervention.

## Author Contributions

**Meiyu Yuan:** conceptualization, data curation, writing – original draft. **Yihong Li:** conceptualization, data curation, investigation. **Zixuan Zhou:** methodology. **Wanting Dai:** methodology. **Xian Cui:** supervision, funding acquisition. **Shuang Bi:** writing – review and editing. **Xiaoxiao Song:** conceptualization, writing – review and editing, project administration, funding acquisition.

## Supporting information


**Figure S1:** (a) pH values of PS, The content of acetic acid produced by PS (b), PS‐SeNPs‐L (c) and PS‐SeNPs‐H (d) at 24 h and 48 h.
**Figure S2:** (a–c) Analysis of KEGG pathway enrichment in different comparative treatment groups.
**Figure S3:** (a) Metabolite VIP bubble chart (HACC‐Se vs. CMC9‐Se). (b) Metabolite clustering dendrogram (CS‐Se vs. COS‐Se).

## Data Availability

The data that support the findings of this study are available from the corresponding author upon reasonable request.
