# Supplementary material for: Investigation Into the Variations in the Interactions Between Different Selenium Sources and Gut Microbiota
Source: Food Sci Nutr. 2026 Jun 4;14(6):e71934. doi: 10.1002/fsn3.71934 (PMC13238918; doi:10.1002/fsn3.71934)
Supplement: Supplementary file 1 — Figure S1: (a) pH values of PS, The content of acetic acid produced by PS (b), PS‐SeNPs‐L (c) and PS‐SeNPs‐H (d) at 24 h and 48 h. Figure S2: (a–c) Analysis of KEGG pathway enrichment in different comparative treatment groups. Figure S3: (a) Metabolite VIP bubble chart (HACC‐Se vs. CMC9‐Se). (b) Metabolite clustering dendrogram (CS‐Se vs. COS‐Se). [file FSN3-14-e71934-s001.docx]

**Supplementary Materials**

**Investigation into the Variations in the Interactions between Different Selenium Sources and Gut Microbiota**

Meiyu Yuan ^a1^, Yihong Li ^a1^, Zixuan Zhou^a^, Wanting Dai^a^, Xian Cui^a^, Shuang Bi^b^, Xiaoxiao Song ^a,^ *

^a^ State Key Laboratory of Food Science and Resources, China-Canada Joint Lab of Food Science and Technology (Nanchang), Key Laboratory of Bioactive Polysaccharides of Jiangxi Province, Nanchang University, Nanchang 330047, China

^b^ School of Food and Health, Beijing Technology & Business University, Beijing 100048, China

*Corresponding author

Xiaoxiao Song

E-mail: songxiaoxiao@ncu.edu.cn (X. Song)

^1^ The first and second authors contributed equally to this work.

**Supplementary Methods**

**2.11** **Metabolomics analysis**

100 μL of fermentation broth sample in a 1.5 mL centrifuge tube, add 400 μL of extraction solution (acetonitrile: methanol= 1:1) containing 0.02 mg/mL of internal standard (L-2-chlorophenylalanine), vortex mixing, ultrasonic extraction for 30 min, and the reaction was carried out for 30 min at -20°C. The sample was centrifuged at 12,000 rpm for 18 min at 4°C, and the supernatant was extracted and blown dry with nitrogen. The residue was redissolved in 100 µL of complex solution (acetonitrile: water=1:1), extracted by low temperature ultrasonication for 5 min (5°C, 40 KHz), centrifuged at 12000 rpm for 12 min at 4°C, the supernatant was pipetted and the sample was analyzed by LC-MS/MS using UHPLC-Q Exactive HF-X. Chromatographic conditions: HSS T3 column (100 mm × 2.1 mm × 1.8 µm). Mobile phase A: 95% water + 5% acetonitrile (containing 0.1% formic acid), mobile phase B: 47.5% acetonitrile + 47.5% isopropanol + 5% water (containing 0.1% formic acid). The flow rate was 0.40 mL/min and the column temperature was:40℃.

Mass spectrometry conditions: Sample mass spectrometry signals were acquired in positive and negative ion scanning modes, with a mass scan range of 70-1050 m/z, sheath gas flow rate of 50 psi, auxiliary gas flow rate of 13 psi, auxiliary gas heating temperature of 425℃, positive-mode ion spray voltage of 3500 V, negative-mode ion spray voltage of -3500 V, and an ion transfer tube temperature of 325℃, and a normalized collision energy of 20-40-60 V cyclic collision energy. The normalized collision energy is 20-40-60 V cyclic collision energy. The normalized collision energy is 20-40-60 V cyclic collision energy. The resolution of the primary mass spectrum is 60,000, the resolution of the secondary mass spectrum is 7,500, and the data are collected in DDA mode.

**Supplementary Figures**

**
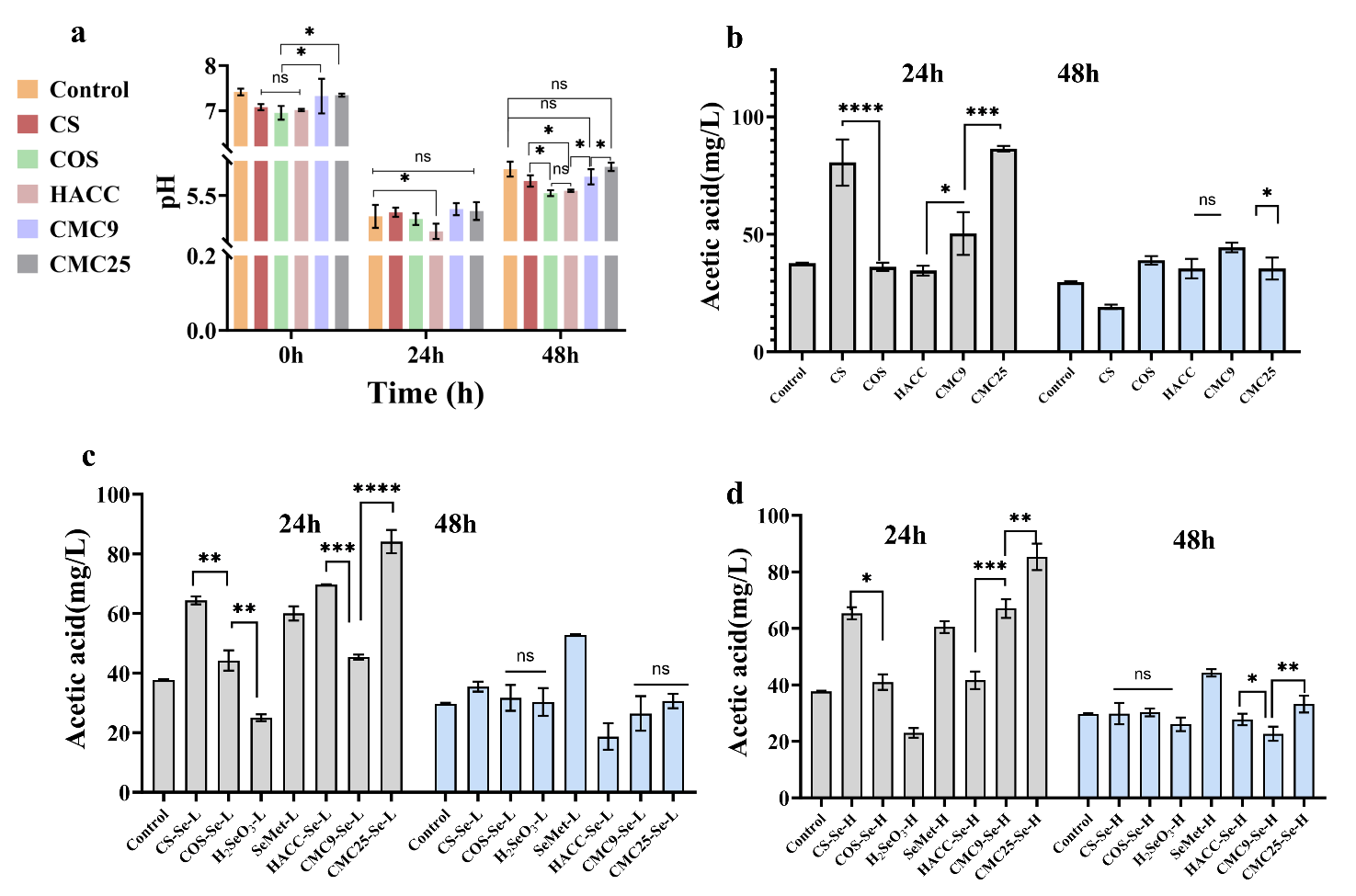
**

**Fig.S1.** (a) pH values of PS, The content of acetic acid produced by PS (b), PS-SeNPs-L (c) and PS-SeNPs-H (d) at 24h and 48h.

**
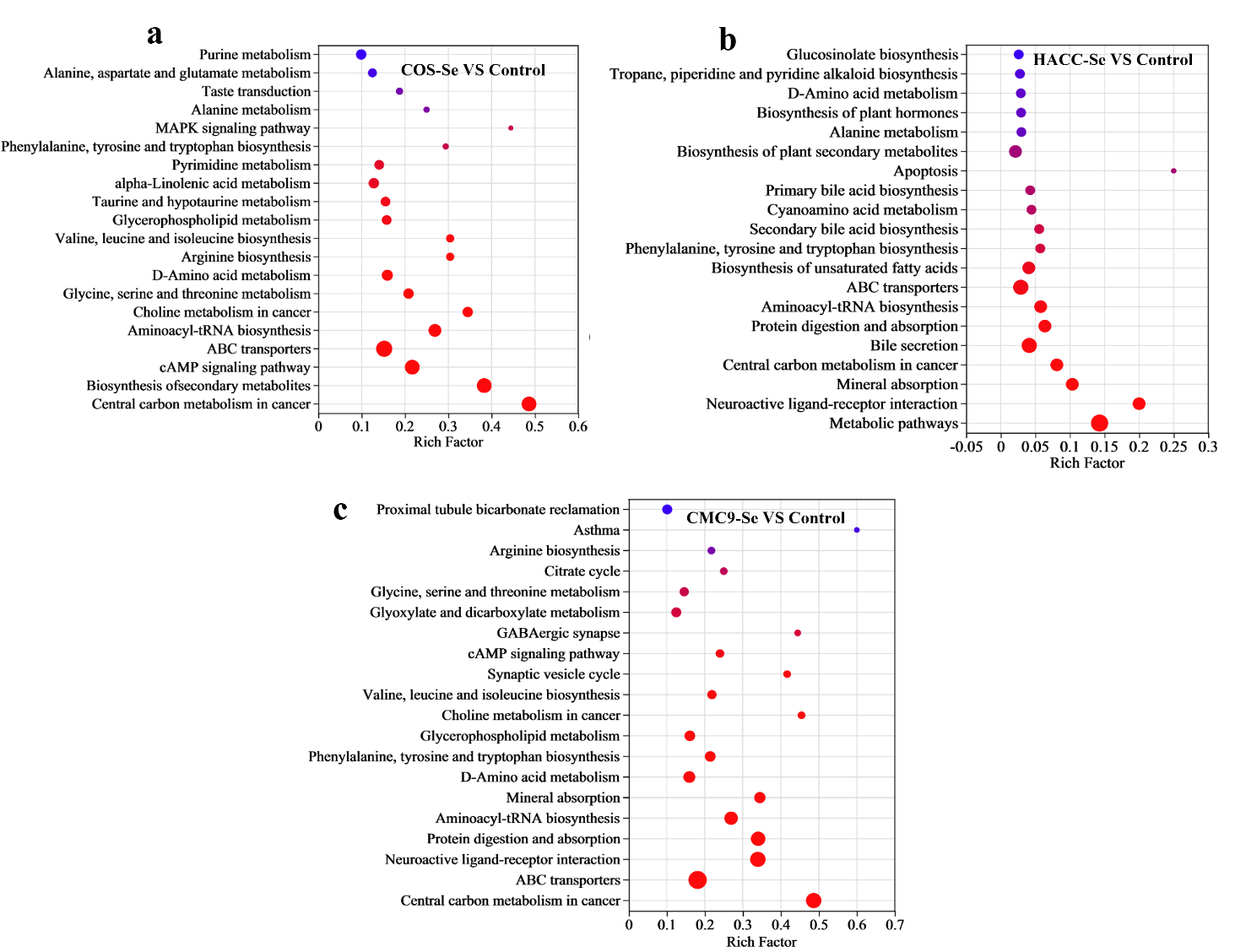
**

**Fig.S2.** (a-c) Analysis of KEGG pathway enrichment in different comparative treatment groups.


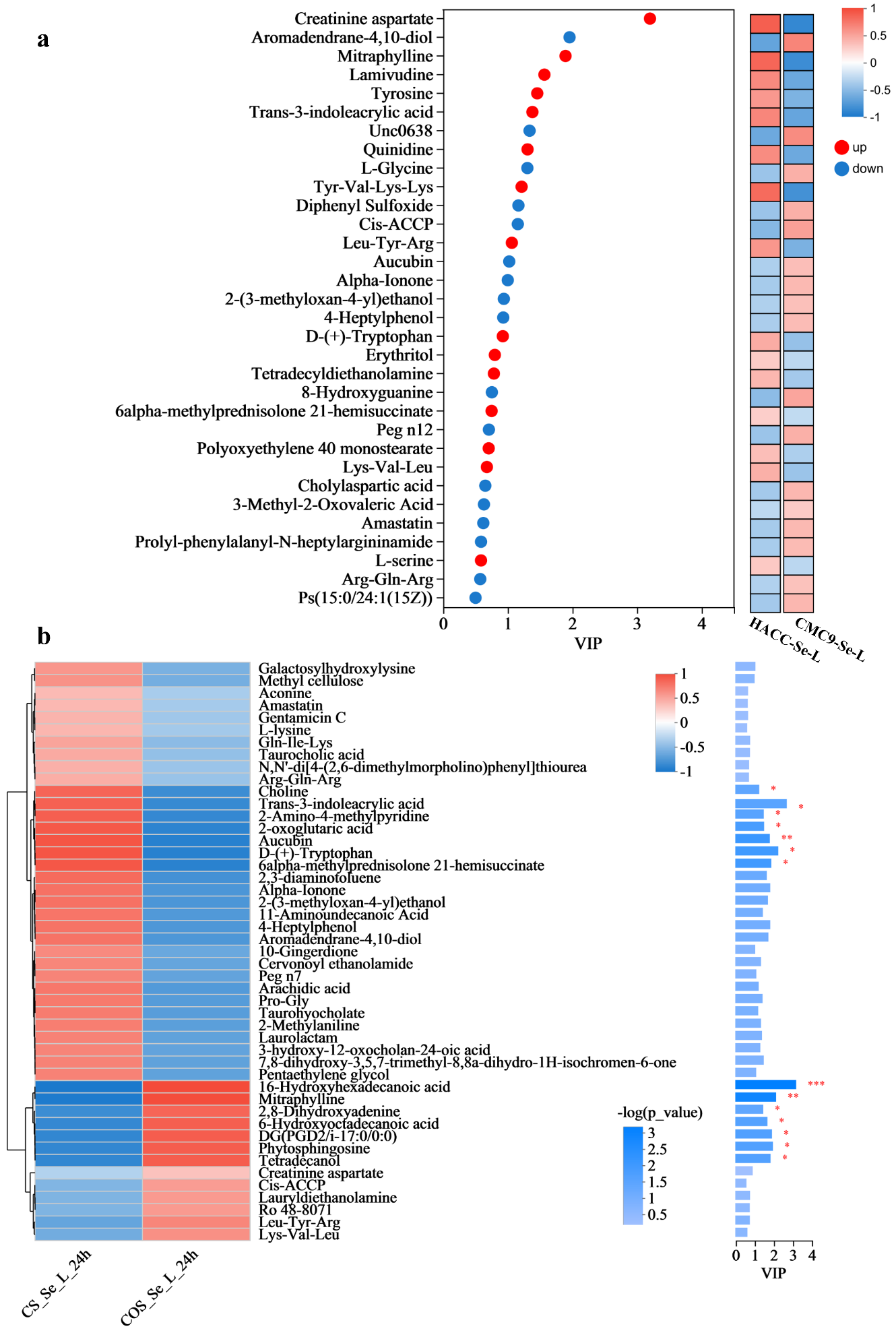


**Fig.S3.** (a) Metabolite VIP bubble chart (HACC-Se vs CMC9-Se). (b) Metabolite clustering dendrogram (CS-Se vs COS-Se)
